# Silencing of sterol glycosyltransferases modulates the withanolide biosynthesis and leads to compromised basal immunity of *Withania somnifera*

**DOI:** 10.1038/srep25562

**Published:** 2016-05-05

**Authors:** Gaurav Singh, Manish Tiwari, Surendra Pratap Singh, Surendra Singh, Prabodh Kumar Trivedi, Pratibha Misra

**Affiliations:** 1 Council of Scientific and Industrial Research-National Botanical Research Institute, Rana Pratap Marg, Lucknow-226001, Uttar Pradesh, India; 2 Banaras Hindu University, Varanasi, Uttar Pradesh, India

## Abstract

Sterol glycosyltransferases (*SGT*s) catalyse transfer of glycon moiety to sterols and their related compounds to produce diverse glyco-conjugates or steryl glycosides with different biological and pharmacological activities. Functional studies of *SGTs* from *Withania somnifera* indicated their role in abiotic stresses but details about role under biotic stress are still unknown. Here, we have elucidated the function of *SGTs* by silencing *SGTL1*, *SGTL2* and *SGTL4* in *Withania somnifera*. Down-regulation of *SGTs* by artificial miRNAs led to the enhanced accumulation of withanolide A, withaferin A, sitosterol, stigmasterol and decreased content of withanoside V in Virus Induced Gene Silencing (VIGS) lines. This was further correlated with increased expression of *WsHMGR, WsDXR, WsFPPS, WsCYP710A1, WsSTE1* and *WsDWF5* genes, involved in withanolide biosynthesis. These variations of withanolide concentrations in silenced lines resulted in pathogen susceptibility as compared to control plants. The infection of *Alternaria alternata* causes increased salicylic acid, callose deposition, superoxide dismutase and H_2_O_2_ in aMIR-VIGS lines. The expression of biotic stress related genes, namely, *WsPR1, WsDFS, WsSPI* and *WsPR10* were also enhanced in aMIR-VIGS lines in time dependent manner. Taken together, our observations revealed that a positive feedback regulation of withanolide biosynthesis occurred by silencing of *SGTLs* which resulted in reduced biotic tolerance.

*Withania somnifera*, commonly known as ashwagandha or Indian ginseng is an important medicinal plant and a rich source of steroidal lactones and terpenoids^1^. Leaf and root extracts of the plant are widely utilized in preparations of various herbal drugs using its biological activities as antioxidative, anti-inflammatory, antidiabetic, antistress, antitumor, hepatoprotective, immunomodulatory, anticonvulsant, antiproliferative, cardioprotective, hypoglycemic, diuretic and hypocholesterolemic^2^,^3^. Withanolides present in the extract of different tissues are the important metabolites having various pharmacological properties. So far, many withanolides have been isolated from *W. somnifera* among which withaferin A and withanolide D were reported to inhibit angiogenesis^4^,^5^. Withanolide A, a major active constituent isolated from *W. somnifera* root predominantly induces axonal outgrowth in normal cortical neurons^6^.

Present knowledge about the withanolide biosynthesis is very limited. Withanolides are C_28_ steroidal lactones which are biosynthesized by 5 carbon precursor isopentenyl diphophate (IPP) and its isomer dimethylallyl diphosphate (DMPP) via cytosolic mevalonate (MVA) pathway and plastid localised methyl-D-erythritol-4-phosphate (MEP) pathway leading to biosynthesis of 24-methylene cholesterol^7^. A series of desaturation, hydroxylation, epoxidation, cyclization, chain elongation and glycosylation steps are involved in withanolide biosynthesis^8^. Of these, glycosylation is one of the most widespread modifications in which simple or complex carbohydrate attaches to the biomolecules. The assembly of carbohydrates is made by a series of enzymatic process of specific glycosyltransferases (GTs), which sequentially transfer the monosaccharide moieties of their activated sugar donor (usually nucleotide donor) to the required acceptor such as lipids and proteins resulting in the formation of a glycosidic bond^9^,^10^.

Sterol glycosyltransferases (*SGT*s) in plants catalyze the transfer of glycon moieties to the sterols and their related compounds to generate steryl glycosides (SGs) or saponins. These glyco-conjugated sterols play very important role in defense of the plant against abiotic stresses and plant pathogen interactions^11^. The saponins produced by oats and tomato have been studied to their potential role in the defense of plants against phytopathogenic fungi^12^. SGs are membrane associated sterols and comprised of a sugar moiety attached to the hydroxyl (-OH) group at C-3 position of a sterol molecule^13^. The hydroxyl group at C-3 position is the most preferred one by *SGT*s for the enzymatic catalysis, followed by 27β-OH position or -OH group present at the side chain of a modified sterol backbone^14^,^15^. *SGT*s have been reported to occur as membrane associated as well as cytosolic. In addition, *SGT*s glycosylate steroidal hormones which function as growth and development regulator in plants^16^,^17^. In *Solanum aculeatissimum*, the accumulation of *SaGT4A* gene for the glycosylation of steroidal sapogenins in response to wounding stress indicates their role in plant defense^18^. *WsSGTL1*, one of the members of *WsSGTs*, has been examined to be involved in response to different abiotic stresses and insect resistance in heterologous expression systems^2^. However, extensive analysis of the role of *SGTs* in the glycosylation of terpenoids and their effect in the plant basal immunity is still lacking.

Virus-induced gene silencing (VIGS) is a quick method for functional analysis by knocking down the gene expression without the need of genetically transforms the plants^19^,^20^,^21^,^22^. Conventional VIGS assays initiate with a large fragment of gene which was converted and modified into small RNAs by the endogenous siRNA-based machinery causing off target gene silencing of the plant. Single artificial miRNA can provide better specificity by minimizing off-target effects^23^. We have developed artificial miRNA and VIGS (aMIR-VIGS) system for the functional characterization of *WsSGTL* genes under biotic stress. In the present study, an efficient protocol has been developed for the down-regulation of phytoene desaturase (*PDS*) gene by a combination of aMIR-VIGS system. We have applied this aMIR-VIGS system for the *SGTLs* members down-regulation of *W. somnifera*. Silenced lines led to significant accumulation of withanolides in leaves as compared to control plants. In addition, *SGTLs* down-regulation of *W. somnifera* affected the early gene transcript of the MVA and MEP pathway. Subsequently, the silneced lines lost their immunity and became susceptible to *Alternaria alternata* infection.

## Results

### Development of efficient aMIR-VIGS constructs

**Figure 1:**
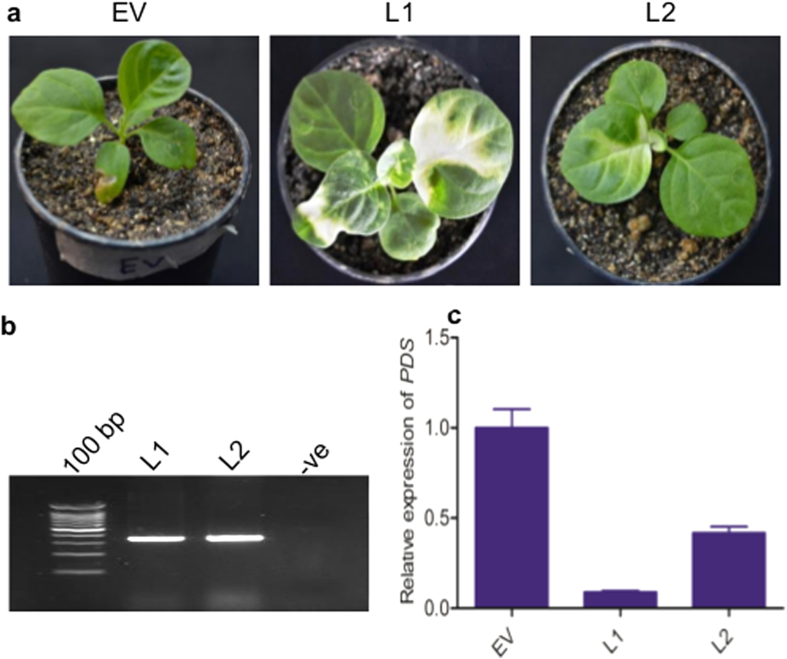
aMIR-VIGS mediated *PDS* gene silencing of *W. somnifera*. (**a**) Phenotypic change in the leaves of silenced plant started from 15–20 DPI after the syringe infiltration of ami*pds*-VIGS construct. (**b**) Positive lines of the plants checked by CP gene specific primer (amplicon size 351 bp). (**c**) *PDS* mRNA levels analysed by qRT-PCR. The expression levels of *PDS* transcripts were normalized to *Actin* gene specific primers. Data are means ± SE of three biological and two technical replicates.

To develop aMIR-VIGS system for *W. somnifera*, we primarily targeted the *PDS* gene by designing amiRNA primer from the conserved region of the gene from closely related species of Solanaceae family (Supplementary Fig. 1a). The ami*pds* construct was prepared by PCR based mutagenesis of miRNA159a of *Arabidopsis thaliana* and cloned into VIGS vector (Supplementary Fig. 1b) Syringe infiltration of this ami*pds*-VIGS construct into *W. somnifera* plants developed bleaching (photobleaching) in the systemic leaves 15 to 20 days post inoculation (DPI) due to *PDS* gene silencing (Fig. 1a). First, the bleached regions were restricted to the veins of the leaves, later the symptoms extended to most of the leaf tissues^24^. The positive lines were also confirmed by coat protein specific primers (Fig. 1b). We have checked the level of *PDS*, by qRT-PCR analysis (Fig. 1c) which concludes that *PDS* mRNA expression was 75 to 90% less in the systemic tissue of ami*pds*-VIGS lines than in control plants (empty vector).

### Silencing of sterol glycosyltransferases

**Figure 2:**
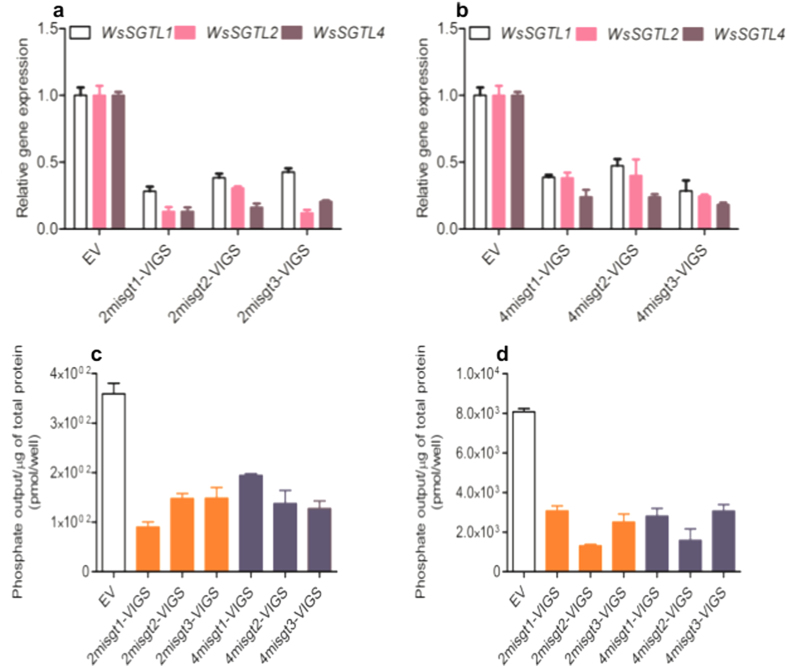
*WsSGTLs* down-regulation by aMIR-VIGS system. (**a,b**) The qRT-PCR showing the relative expression level of *WsSGTLs* members in 2mi*sgt*-VIGS and 4mi*sgt*-VIGS down-regulated lines as comparision to control (EV) with *WsSGTL1, WsSGTL2* and *WsSGTL4* gene specific primers. The expression levels of *WsSGLTs* transcript were normalized to *Actin*. (**c,d**) The relative *WsSGTLs* enzyme activity from the total protein of silenced lines by using stigmasterol and solenidine as accepter molecule respectively. Data are means ± SE of three biological and two technical replicates.

In order to investigate the role of *SGTLs* in *W. somnifera*, initially three different amiRNAs were designed from conserved sequences of *WsSGTL1*, *WsSGTL2* and *WsSGTL4*, named as, 2mi*sgt*, 4mi*sgt* and 6mi*sgt* (Supplementary Fig. 2). To examine their silencing efficiency, amiRNAs were first cloned into pBI121 (Supplementary Fig. 3a) and then transformed into leaves of 3-weeks-old plants (Supplementary Fig. 3b). After 48 h and 72 h, qRT-PCR of 2mi*sgt*-pBI121 and 4mi*sgt*-pBI121 showed better silencing efficiency against members of *SGTLs* expression than 6mi*sgt*-pBI121 (Supplementary Fig. 3c,d). Further, 2mi*sgt* and 4mi*sgt* amiRNAs were utilized to clone into VIGS vector for the development of aMIR-VIGS system named as 2mi*sgt*-VIGS and 4mi*sgt*-VIGS (Supplementary Fig. 4a,b). The aMIR-VIGS construct of *WsSGTLs* members were transformed into leaves of 3-weeks-old plants. After 4 weeks of transformation, positive aMIR-VIGS lines were selected through PCR of CP specific primers (Supplementary Fig. 4c). The qRT-PCR were performed by using *SGTL1, SGTL2* and *SGTL4* gene specific primers and observed that their transcript level was decreased in the aMIR-VIGS plants. Interestingly, both aMIR-VIGS constructs showed almost same level of down-regulation of *SGTLs* members (Fig. 2a,b) as compared to control plants.

We have performed comparative nonradioactive sterol glycosyltransferase assay in the aMIR-VIGS lines of *W. somnifera*^25^ and control plants. Stigmasterol and solanidine were taken as acceptor molecule which have free 3β-OH moiety group. Accepter molecules were modified by donor substrate, uridine 5′-diphosphoglucose (UDP-glucose) with the help of SGT enzyme. This method takes advantage of a specific phosphatase to remove inorganic phosphate quantitatively from the leaving nucleotide diphosphate, such as UDP or GDP, of glycosyltransferase reactions. Total phosphate output/well in the protein reaction of 2mi*sgts*-VIGS and 4mi*sgts*-VIGS lines decreased significantly (upto 90%) as compared to control plants (Fig. 2c,d). This confirms that due to down-regulation of members of *WsSGTs*, silenced lines posses lesser SGT activity due to decreased expression of the genes.

### Down-regulation of *WsSGTLs* modulate withanolides and phytosterols

**Figure 3:**
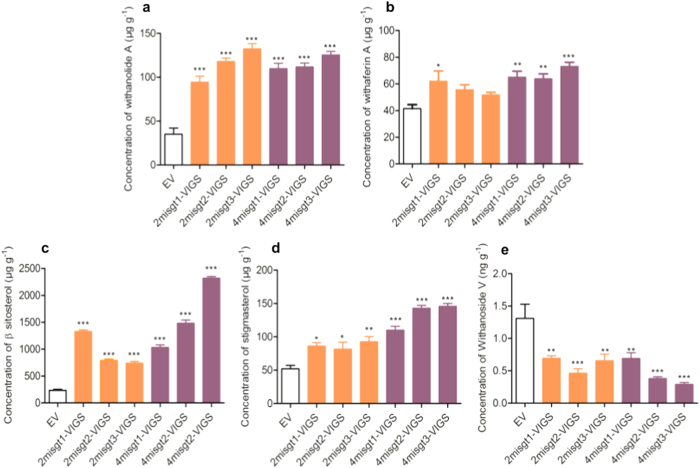
Withanolide profiling of control and silenced lines. Relative quantification of HPLC analysis of withanolide A, withaferin A, sitosterol stigmasterol and withanoside V in the 2mi*sgt*-VIGS and 4mi*sgt*-VIGS down-regulated lines of *W. somnifera* as compare to EV. Data are means of three biological replicates plants. Asterisk indicate the significance level between control and down-regulated lines (*P ≤ 0.05, **P ≤ 0.01, ***P *≤* 0.001) by Dunnett test.

To investigate the role of *SGTL* members in withanolide biosynthesis, we quantified withanolide A, withaferin A, withanoside V sitosterol and stigmasterol contents in leaves of each aMIR-VIGS plant after 40 DPI of transformation through high-performance liquid chromatography (HPLC) (Supplementary Fig. 6a–i). Quantitative estimation showed that withanolide A (upto 3.7 fold) and withaferin A (upto 1.7 fold) content significantly increased in 2mi*sgt*-VIGS and 4mi*sgt*-VIGS aMIR-VIGS lines as compared to control plants (Fig. 3a,b). In addition, concentration of sitosterol (upto 9.9 fold), stigmasterol (2.8 fold) also increased significantly in silenced lines (Fig. 3c,d). Analysis of withanoside V showed a significant reduction (approximately 45% to 77%) in 2mi*sgt*-VIGS and 4mi*sgt*-VIGS lines after down-regulation of *SGTLs* (Fig. 3e).

**Figure 4:**
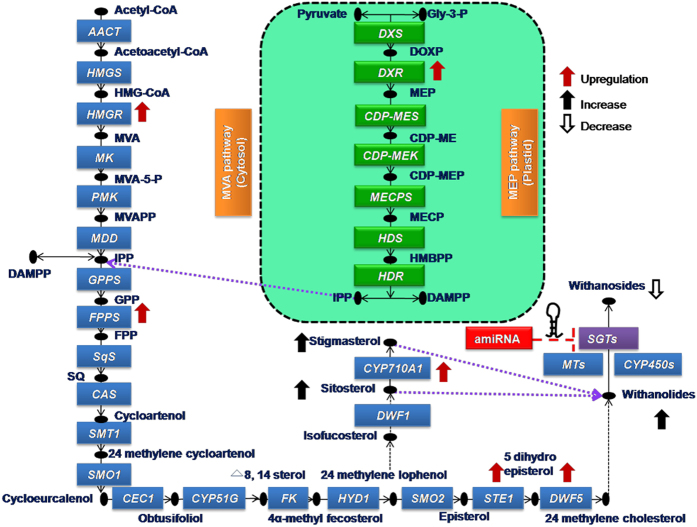
Isopreniod biosynthesis pathway in the plants. Model for MVA and MEP pathway (in green box) in *W. somnifera*. Solid one headed arrow indicates the single step irreversible while two headed for reversible reaction. Dashed purple arrow indicates several steps of reactions. All important enzyme of the pathway has been listed in blue box. Repression of *SGTLs* members by aMIR-VIGS system is represented by red dashed lines. Down-regulation of *WsSGTLs* members causes the increased in withanolides contents (black upward arrow) while decrease in the concentration of glycowithanolide (downward arrow). The up-regulation of important intermediate genes shown by upward red arrows indicates the increased withanolide biosynthesis in silenced lines.

Analysis was carried out to study whether the down-regulation of members of *WsSGTL* gene family affected the expression of genes involved in intermediate steps of the withanolide biosynthesis. To study this, transcript level of *WsHMGR, WsDXR, WsFPPS, WsCYP710A1, WsDWF5* and *WsSTE1* genes involved in MVA pathway were analysed by qRT-PCR in the aMIR-VIGS lines and control plants (Supplementary Fig. 7). There was a significant up-regulation (approx 3 fold) of *WsCYP710A1* expression which might be responsible for the increase in the concentration of sitosterol and stigmasterol in silenced lines. Up-regulation of *WsSTE1* (approx 3 fold) and *WsDWF5* (approx 3.5 fold) was observed which might be responsible for increased withanolide A and withaferin A content in silenced lines. The expression of intermediate genes, *WsHMGR*, *WsDXR*, *WsFPPS* in aMIR-VIGS lines were also up-regulated upto 2.8, 10 and 2.9 fold, respectively. Our metabolite content and gene expression analysis conclude that down-regulation of *SGTLs* modulates the accumulation of withanolides by increasing expression of genes involved in MVA pathway (Fig. 4).

### Silencing of *WsSGTLs* affect growth and development of plant

**Figure 5:**
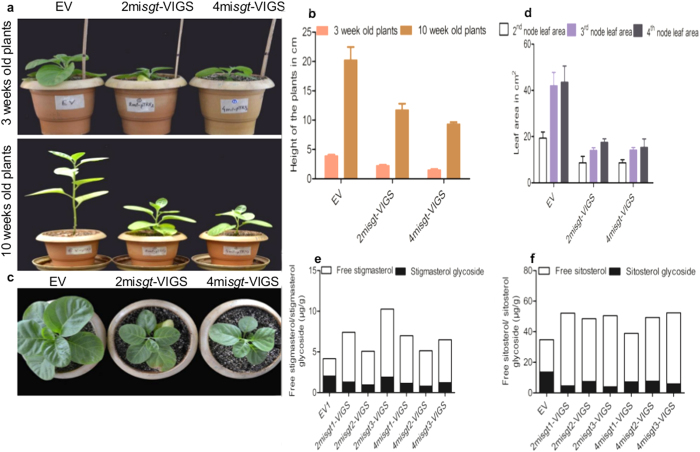
Silencing of *WsSGTLs* members affect growth of the *W. somnifera*. (**a–d**) Down-regulation of *WsSGTL1*, *WsSGTL2* and *WsSGTL4* of *W. somnifera* significantly affects the height and leaf area of the plants but no change in the number of nodes and internodes. (**e,f**) ratio of free phytosterol to their respective glycoside. Height data are means ± SE of three different plants and leaf area from three leaves of different plants at same node. HPLC data are the means ± SE of three technical replicate.

The down-regulation of *WsSGTLs* resulted delay in the normal growth of aMIR-VIGS lines. After 3 weeks of transformation, plant height and leaf area of the silenced lines was measured. The height of the aMIR-VIGS lines was significantly shorter than control plants. This difference was maintained even after 10 weeks of growth (Fig. 5a,b), however, there was no change in the number of nodes and internodes. Total leaf area of the silenced lines was lesser than the control plants (Fig. 5c,d). These results suggest that the ratio of free sterol and its glycosylated forms may work as signalling molecules and regulate the growth of the plants. For the confirmation of this hypothesis, we have measured the ratio of free sitosterols and stigmasterol vs its glycosylated forms by acid hydrolysis^26^. The ratio of free phytosterol vs glycosylated phytosterols was higher in silenced lines than control plants (Fig. 5e,f). This experiment gave us indication of sterol imbalance in VIGS lines of *W. somnifera* and might be one of the reasons of shorting the height and leaf area of plants.

### Suppression of *WsSGTL*s increases disease susceptibility

**Figure 6:**
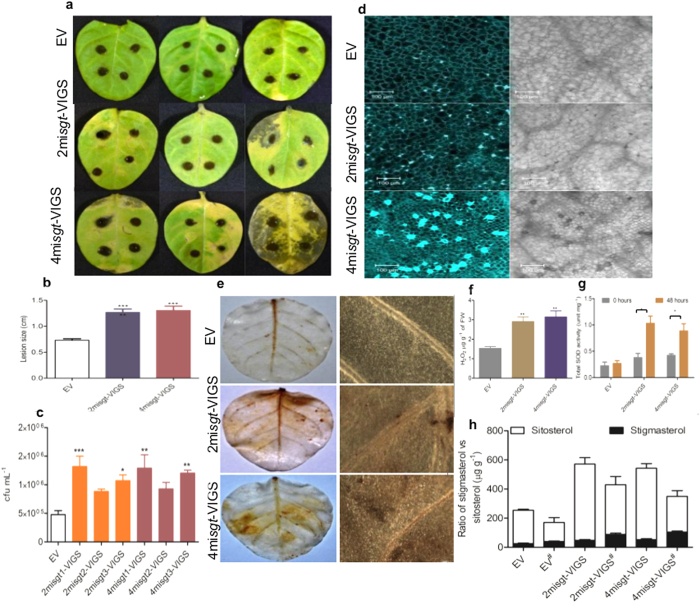
Down-regulation of members of *SGTLs* increases disease susceptibility of *W. somnifera*. (**a**) Infection of *W. somnifera* with *A. alternata* strains, symptoms in the leaves in 7 days after inoculation. (**b**) Statistical data of lesion size 7 days after infection. For lesion size, the data are means ± SE of two independent experiments representing six biological replicates. Asterisk indicates a significant difference from the control (unpaired Student’s t-test; ***P < 0.05). (**c**) Total colony forming unit (cfu) count of the aMIR-VIGS plants leaves after 7 days infection of *A. alternata*. Data are the means of four biological replicates of each silenced lines. Asterisk indicates the significant level at *P ≤ 0.05, **P ≤ 0.01, ***P ≤ 0.001 by Dunnett test. (**d**) Aniline blue staining in the leaf of *W. somnifera* after the infection of *A. alternata* at 7 dpi at 20X magnifications on confocal microscope. Bars = 100 μm. (**e,f**) Hydrogen peroxide accumulation in leaves of *W. somnifera* were analysed after 48 h of infection by the microscopic (DAB staining, under light microscopy at 40 X magnification) and spectroscopic analysis. Data are the means ± SE of three different biological replicates. Asterisk indicates the significant difference from the control (unpaired Student’s t-test, **P ≤ 0.05). (**g**) Total SOD has been measured after 48 h infection of *A. alternata* in control and silenced lines of *W. somnifera*. Data are expressed in mean ± SE of three different biological replicates. Asterisk indicates the significant difference from the control (unpaired Student’s t-test, *P ≤ 0.05). (**h**) Stigmasterol/sitosterol ratio in control and silenced lines before and after 7 days *A. alternata* infection. Data are the means ± SE of three biological replicate samples. (*) asterisk is the indication of biotic stress condition.

To determine the disease susceptibility of 2mi*sgt*-VIGS and 4mi*sgt*-VIGS line leaves against *A. alternata*, experiments were conducted under optimum growth conditions for the pathogen infection. First upper leaves of the silenced lines and control plants were inoculated with the fungal spore suspension. The severe necrotic symptoms were observed 7DPI of infection (Fig. 6a). After the infection of *A. alternata*, area of the lesion (Fig. 6b) and chlorosis in the leaves of 2mi*sgt*-VIGS and 4mi*sgt*-VIGS lines was much higher than control plants. The extracts isolated from the infected leaves were used for total colony forming unit count (cfu) which was significantly higher in silenced lines than control plants (Fig. 6c).

Furthermore, callose deposition in the leaves of infected plants is another important parameter to check disease susceptibility against the biotic stress. After 7DPI of *A. alternata* infection, the leaf of the silenced lines and control plants was stained with aniline blue. Under confocal microscope, the higher amount of callose deposition was observed in the leaves of 2mi*sgt*-VIGS and 4mi*sgt*-VIGS lines as compared to the leaves of control plants (Fig. 6d). In addition to evaluate *A. alternata* induced disease symptoms, microscopic studies were conducted in down-regulated lines for cellular and intercellular reactive oxygen intermediates (ROIs). Hydrogen peroxide (H_2_O_2_) was detected by non-fluorescent (3, 3′-diaminobenzidine [DAB]) staining method. Microscopic analysis after 48 h infection revealed a strong dark brown precipitation in the leaves of 2mi*sgt*-VIGS and 4mi*sgt*-VIGS lines whereas no such precipitation was observed in the leaves of control plants (Fig. 6e). This was further accomplished by quantitative analysis of H_2_O_2_ where it increased (upto 1.8 fold) in the leaves of aMIR-VIGS lines after 48 h of fungal infection (Fig. 6f). We also quantified SOD level at 0 h and after 48 h post *A. alternata* infection in 2mi*sgt*-VIGS, 4mi*sgt*-VIGS lines and control plants. At 0 h, no significant differences were observed in the level of SOD, whereas, SOD level significantly increased upto 1.1, 2.7 and 2.1 for EV, 2mi*sgt*-VIGS and 4mi*sgt*-VIGS lines, respectively after 48 h of *A. alternata* infection, (Fig. 6g). The ratio of stigmasterol/sitosterol also maintains the innate immunity by increasing the disease susceptibility of the plant leaves. We have measured the stigmasterol/sitosterol ratio in plant before and after 7 days of *A. alternata* infection. After 7 days of stress, the concentration of sitosterol was decreased whereas, stigmasterol concentration increases in silenced lines and control plants. Although, stigmasterol/sitosterol ratio were higher in silenced lines than control plant (Fig. 6h). These results indicated that after down-regulation of members of *WsSGTLs*, the plants lose their basal immunity and became more prone to infection with *A. alternata*.

### Salicylic acid level increases the expression of defence related genes in silenced lines

**Figure 7:**
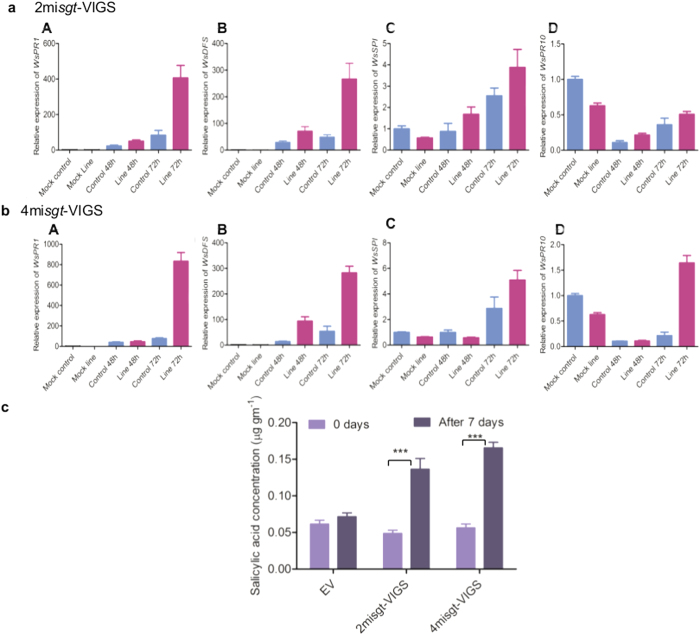
Effect of *A. alternata* infection on salicylic acid content and defense related genes expression of *W. somnifera*. (**a,b**) Expression level of different defense genes such as (A), *WsPR1* (B), *WsDFS* (C), *WsSPI* have significantly increased while (D), *WsPR10* showed delayed over-expression in silenced lines after 48 h and 72 h of fungal infection as compare to Mock, taken as control. (**c**) Salicylic acid quantification in the silenced plants leaves before and after 7 days infection of *A. alternata* from 40 DPI old plants. Data are expressed in the mean ± SE of three different biological replicates. Asterisk indicates the significance level as compare to control (unpaired Student’s t-test, at ***P *≤* 0.05).

To investigate the contribution of defense related genes against *A. alternata* infection in silenced lines, real time expression analysis of some important genes, namely, *WsPR1, WsDFS, WsSPI* and *WsPR10* was performed on the basis of earlier published transcriptome analysis^27^. The maximum enhancement in expression of *WsPR1* was found upto 406 to 833 fold after 72 h of fungus infection in 2mi*sgt*-VIGS and 4mi*sgt*-VIGS respectively, whereas, upto 83 fold in EV as compared to mock plant (Fig. 7a[A] and 7b[A]). The increase in the expression level of *WsDFS* and *WsSPI* was also similar to the expression level of *WsPR1* (Fig. 7a[B-C] and 7b[B-C]). Finally, it was concluded that after *A. alternata* infection, the expression of defense related genes in silenced lines of *W. somnifera* was highly up-regulated as compared to control plants.

The salicylic acid (SA) signalling cascades are the master regulator of defense process in plants which control the gene expression of large number of genes. Modulation of defense related gene expression encouraged us to study the possible role of SA. To know this, we measured SA levels before and after 7 days of *A. alternata* infection in aMIR-VIGS lines and control plants. Before the infection of *A. alternata*, no significant difference in the level of SA was observed in silenced lines and control plants. However after infection with *A. alternata*, SA level increased upto 3 fold in 2mi*sgt*-VIGS and 4mi*sgt*-VIGS lines as compared to control (Fig. 7c, Supplementary Fig. 10a–d). Altogether, these observations indicated that increased level of SA was responsible for modulation of defence related gene expressions which ultimately altered the basal disease resistance in aMIR-VIGS lines.

## Discussion

*W. somnifera* is a medicinal plant rich in sterols, sterol glycosides and steroidal lactones which play a crucial role in adaptation of the plants to stress conditions^28^. In our previous studies, we reported that over-expression of *WsSGTL1* in heterologous systems enhances germination, salt tolerance, heat tolerance, cold tolerance and insect resistance as compared to control plants^2^,^28^. In the present report, silencing of members of *WsSGT*s was done using the amiRNA technology^29^. However, Virus-induced gene silencing (VIGS) is also a quick method for knocking down the gene expression^21^. We designed artificial miRNA and developed efficient aMIR-VIGS system for the functional analysis of *SGTLs* members in *W. somnifera*.

The gene silencing by VIGS within a plant is highly dependent on the environmental factors and method of inoculation^30^,^31^,^32^. The marker gene used for the initiation of virus symptoms is *GFP* or *PDS*^24^,^33^. Gene silencing of *PDS* gene of *W. somnifera* through ami*pds*-VIGS causes suppression of carotenoid biosynthesis where the affected plants became susceptible to photobleaching^20^,^24^,^34^,^35^. This protocol of aMIR-VIGS system has been used for the down-regulation of *WsSGTs* members to obtain a comprehensive understanding of their role in plants.

The members of *WsSGTL* are important modifying enzymes which participate in isoprenoid biosynthetic pathway, specifically in the glycosylation of phytosterols and withanolides. We have developed silenced lines of *W. somnifera* using aMIR-VIGS system. The down-regulation of members of *WsSGT*s was confirmed by comparative qRT-PCR analysis and SGT enzymatic assay of aMIR-VIGS lines and control plants. *WsSGTLs* down-regulated lines accumulated high amount of withanolide A, withaferin A, sitosterol and stigmasterol, whereas, as expected less amount of withanoside V in the leaves of silenced plants. It has been already reported that over-expression of glycosyltransferase genes leads to a significant increase in their respective glucosides^36^,. Recently, it was cleared that after over-expression of *WsSGTL1* gene in homologous system increased the production of glycowithanolides^37^. To develop a better understanding of the effect of down-regulation of *WsSGTLs*, qRT-PCR of *WsHMGR, WsDXR, WsFPPS, WsCYP710A1, WsSTE1* and *WsDWF5* (genes of MVA pathway) was performed. From this analysis, it was concluded that *WsSGTLs* down-regulation positively affected the upstream genes of MVA pathway (Fig. 4, Supplementary Fig. 7). Recently, it was published that *WsSQS* leads to positive feedback and negative feed-forward regulation of sterol biosynthetic genes. Down-regulation of *WsSQS* significantly modulates the expression of MVA pathway genes located upstream and downstream of *SQS*^5^. From these results, we could conclude that the accumulation of withanolides and increased expression of biosynthetic pathway genes were positively affected by the down-regulation of members of *WsSGTLs*.

In addition, *WsSGTLs* down-regulated lines also showed phenotypic changes which suggested that *Ws*SGTL*s* activity may be affecting the growth hormone signalling pathway in plants. In the silenced lines, height and leaf area were significantly shortened as compared to control plants (Fig. 5a–d). It was already proved that one members of *WsSGTL* gene family i.e., *WsSGTL1* over-expression causes the alteration in phenotype of the plants such as enhanced growth and expansion of leaves^37^. Many reports suggested that glycosylation of important hormones, brassinosteroids and strigolactone was being carried out by the members of *SGT*s in plants^13^,^38^. The SDG8i glucosyltransferase and *UGT74D1* were capable of influencing the biological activity of plant hormones such as strigolactone -oxindole-3-acetic acid (OxIAA) via glycosylation^38^. Gene mutants of *UGT80B*1and *SMT1* have revealed roles of sterols modifications in embryo, vein, shoot and root patterning, cell expansion, polarity and proliferation, fertility, cellulose level maintenance, gravitropism, and hormone signalling^39^,^40^. After HPLC analysis of silenced lines it was concluded that the ratio of free phytosterol and their respective glycoside higher than control plants (Fig. 5e,f). It may be concluded that *WsSGTLs* down-regulation causes imbalance of glycosylated and nonglycosylated sterol which results in phenotypic changes of silenced lines. Besides this, the down-regulation might be affecting the signalling pathway of hormones which hinders the growth of plants.

Earlier reports showed the role of *WsSGTL1* in response to abiotic stresses in heterologous system^2^,^28^ but no report is available regarding biotic stress. It was already reported that expression of *SGTs* were differentially modulated in different organs in response to external stimuli such as treatment of SA and methyl jasmonate in *W. somnifera*^41^, suggesting their role in defense. Here, we report that the down-regulation of *WsSGTLs* leads to increased susceptibility of plants against the infection of *A. alternata*.

Necrotrophic fungus, *A. alternata*, produces an AAL toxin (TA and TB) that causes necrosis in more than 200 species of Solanaceae family^42^,^43^. In biotic stress experiments, the leaves of silenced lines have 2–3 fold higher total cfu mL^−1^ count than control plant leaves. Callose also plays an important role in plant defense during interactions with pathogens. It rapidly synthesized and deposited just beneath the sites of attempted pathogen penetration^44^,^45^,^46^. The susceptibility was further evident by the massive callose deposition in the leaves of aMIR-VIGS lines as compared to control plants after one week of *A. alternata* infection. Further, we quantified H_2_O_2_ and SOD accumulation in silenced lines and control plants after the infection. It was reported earlier that SOD and H_2_O_2_ production plays a crucial role in restricting the growth of pathogen during hypersensitive response^47^,^48^,^49^,^50^,^51^. In the present study, *WsSGTLs* silenced lines accumulated high amount of H_2_O_2_ and SOD after the fungus infection. Several reports are available which emphasizes that exposure of stress resulted in increase in SOD activity due to increase in the ROS and H_2_O_2_ levels^52^,^53^. Since the high SOD level causes the high rate of H_2_O_2_ production in cell^54^. After pathogenic infection, plants induced array of metabolites change that potentially contributed in the susceptibility of the plants against pathogen infection. The conversion of sitosterol to stigmasterol is one of the significant marker of susceptibility against pathogenic infection and stigmasterol/sitosterol ratio become rise^55^,^56^. After the *A. alternata* infection, the conversion of sitosterol to stigmasterol was reported in the *WsSGTLs* silenced lines and control plants. This conversion was little high in the aMIR-VIGS lines than control plants. Resultant, the C22 desaturation of sterol was high in the silenced plant membrane after fungal infection and plants showed more disease susceptibility.

The level of SA was estimated before and after *A. alternata* infection in *WsSGTLs* silenced lines and control plants. SA plays important role in plant defense and involved in the activation of defense responses against biotrophic and hemi-biotrophic pathogens as well as in the establishment of systemic acquired resistance (SAR)^57^,^58^. It was also reported that programmed cell death was directly induced by level of SA^58^. This was one of the reasons of nercosis and callose deposition in the leaves of silenced lines in our study. Several previous reports demonstrated that the level of SA induced the expression level of PR proteins and other defense related genes^59^,^60^,^61^,^62^. To check this hypothesis, qRT-PCR of *WsPR1, WsDFS, WsSPI* and *WsPR10* was performed in silenced and control plants and compared with the mock plants. Expression analysis showed up-regulation of these genes in time dependent manner^63^,^64^.

Finally, it may be concluded that after down-regulation of *SGTL1, SGTL2* and *SGTL4* genes of *W. somnifera*, withanolide contents were increased, whereas withanoside content was decreased. Also, this modulation was analysed with the increase in the expression of some important intermediate genes of MVA pathway. Silencing of *WsSGTL* members, modulation was observed in the free and glycosylated phytosterols which made the phenotypic variations in plants. Due to this modulation of withanolides,withanosides and ratio of nonglycosylated vs glycosylated phytosterols the plants might have lost their first line of defense. Further, the infection of *A. alternata* increased levels of SOD, H_2_O_2_ and SA significantly in down-regulated lines as compared to control plants. Higher level of SA also modulated the expression of *WsPR1, WsDFS, WsSPI* and *WsPR10* genes of plant defense system. Altogether, we could demonstrate that the members of *WsSGTLs* play a significant role in maintaining the metabolic balance of the cell which facilitated the proper growth and defense system of the plants.

## Methods

### Preparation of artifitial miRNA

Expression of amiRNAs requires a mature miRNA backbone sequence, of which the stem region was replaced with desired sequences. A 545-bp fragment containing the entire sequence of the *Arabidopsis* miR159a was cloned into pTZ57R/T vector by PCR amplification using primers miR159-F1 (5′-ATATCTCCTTCATAGCTCTAATG-3′), miR159R1 (5′-AAATAACACGCTAAACATTGCTTCG-3′) and utilized for mutagenesis to yield amiRNA for *WsPDS* and members of *WsSGTLs* using oligonucleotide primers. For the preparation of these oligonucleotide primers, *PDS* sequences of closely related species of Solanaceae family and *SGTLs* sequences of *W. somnifera* were downloaded from National Centre for Biotechnology Information (NCBI) database. 21 bp conserve region for *PDS* and *WsSGTLs* were selected by multalin online tool (Supplementary Figs 1 and 2). Three conserve sequences of *WsSGTLs* 2mi*sgt*, 4mi*sgt*, 6mi*sgt* and one *PDS* named ami*pds* (Supplementary Table 1) were selected on the basis of their specificity for the target. Three constructs have been prepared in pBI121 by using pTZ-miRNA159a as template named 2mi*sgt*-pBI121, 4mi*sgt*-pBI121 and 6mi*sgt*-pBI121 (Supplementary Fig. 3a) and silencing efficiency checked by syringe infiltration of these construct in leaves of the 3–4 week old plants after germination.

### aMIR-VIGS construct preparation

Mutagenesis of pTZ57R/T-premiR159a construct was performed by PCR by using efficient 2mi*sgt*, 4mi*sgt* and ami*pds* oligonucleotide primers. PCR product (247 bps) and pTRV vector were digested with Xba I and Sac I restriction enzymes (Fermentas, Fast Digest) and ligated with 1 μL of T4 DNA ligase (200 CEU μL^−1^ Fermentas). Ligated constructs were transformed into DH5α strain of *E. coli* and colonies were checked by amplification of 562 bp replicase, 351 bp coat protein and 247 bp amiRNA specific primers and further confirmation of positive construct was done with sequencing.

### Plant growth and agroinfiltration

*W. somnifera* plants were grown at 28 °C under 16-h-light and 8-h-dark conditions in growth chamber. For VIGS assay, gene of interest (GOI)::TRV2 (2mi*sgt*-VIGS, 4mi*sgt*-VIGS and ami*pds*::TRV2) constructs were transformed into GV3101 strain of *Agrobacterium*. Positive transformants were selected through colony PCR (Supplementary Fig. 4a,b) and grown on Luria-Bertani agar medium (50 mg mL^−1^ kanamycin, 50 mg mL^−1^ gentamycin and 25 mg mL^−1^ rifampicin). Two days before infiltration, 5-mL primary cultures of *Agrobacterium* strains were inoculated from single colonies on plates and grown for 16 h at 28 °C. 20 mL secondary culture was inoculated from the primary culture and grown for 5–6 h for obtaining OD_600_ 0.4. The culture was harvested by centrifugation at 3000 × g at room temperature for 5 min. Cell pellet was resuspended in same volume of induction buffer, 10 mM MES (pH 5.5), 200 μM acetosyringone and incubated for 3–4 h at 28 °C at 80 rpm. Then pallet the culture again at 3000 × g and resuspended in the infiltration buffer containing 5 mM MES (pH 5.5) and OD_600_ was adjusted to 0.8–0.9. TRV1 + GOI: TRV2 cultures were mixed at 1:1 (v/v) ratio. A needleless syringe was used to inoculate the abaxial side of the lower leaves with TRV1 + GOI::TRV2 mixture of *Agrobacterium* culture. Infiltration of TRV1::TRV2 in the plants served as control (empty vector). The inoculated plants in the greenhouse were maintained at 21 ± 2 °C until the observation period was achieved.

### RNA isolation and RT-PCR

Total RNA was extracted from upper leaves of 3-weeks-infiltered plants by using the RNA isolation kit (Sigma-Aldrich) and treated with RNase-free DNase I (Ambion). First-strand complementary DNA was synthesized using 5 μg of total RNA with oligo (dT) primer (Fermentas). The qRT-PCR was done in the StepOnePlus Real-Time PCR System (Applied Biosystems 7500). Primers used for qRT-PCR are listed in Supplementary Table S2. Three independent biological replicates and for each biological replicate, two technical replicates were analyzed by qRT-PCR analysis.

### SGT assay

The SGT activity was measured by the Glycosyltransferase activity kit (R&D system/USA). The assay was carried out by detection of released inorganic phosphate using Malachite Green reagent^25^. Total protein was isolated by the ReadyPrep™ Protein Extraction Kit (Bio-Rad) and quantified by Bradford method^65^. For the SGT enzyme assay, phytosterols [stigmasterol (Sigma), solanidine (Sigma)] and UDP-glucose (Sigma) were used as accepter and donor substrates, respectively. The amount of phosphate removed from the acceptor molecule was calculated use via phosphate standard curve (Supplementary Fig. 5).

### Biochemical Analysis by HPLC

The extraction procedure of withanolide (withanolide A and withaferin A) and glycowithanolide (withanoside V) has been followed by the protocol^66^. Qualitative and quantitative analysis of withanolides and withanoside were performed by HPLC-PDA with a Shimadzu (Japan) LC-10A system comprising LC-10AT dual-pump system, a SPD-10A PDA detector (operated at 227 nm), and Rheodyne injection valve with 20-μL sample loop. Compounds were separated on a RP-C18 column (Merck) (4.6 mm × 250 mm, 5-μm pore size) protected by a guard column containing the same packing. 1 mg mL^−1^ stock solutions of withaferin A, withanolide A and withanoside V (Sigma) were subsequently diluted to prepare solutions with concentrations in the range of 0.5 mg mL^−1^ to 50 mg mL^−1^ for preparation of standard curve. The sitosterol and stigmasterol estimation was carried out by following the protocol^2^. Ratio of these phytosterols of the plant leaves were checked by before and after acid hydrolysis by HCL^26^. For the quantification of SA, 200 mg sample was crushed into 1 mL of methanol and the volume was made to 2 mL. Mobile phase prepared from 20 mM phosphoric acid (A) (pH 2.0 adjusted) and acetonitrile (B) added in 50:50 ratio (A:B). Quantification was performed at 220 nm with PDA detector at 25 °C column temperature and 20 min RT with the flow rate of 1 mL min^−1^.

### Biotic stress assay with *A. alternata*

Leaves of *W. somnifera* were surface sterilized with 1% NaOCl (v/v) and placed at blotting paper. The leaves were transferred into petriplates containing 0.8% agar. For pathogenic assay, 0.5% gelatine was freshly prepared to which 7–10 days old fungal spores were suspended to make the final fungal spore concentration of 10^6^ cfu mL^−1^. Each leaf was treated with 5 μl of spore suspension at four different locations on the adaxial surface of the leaves. Control and pathogen infected leaves were kept at 25 °C under 70% relative humidity and 16 h of diurnal light. After 7 days of infection, leaves were sampled for measurement of lesion diameter and for total spore count.

### Callose staining

For callose deposition assay, spore suspension sprayed on the leaves of control and transgenic plants with the help of an atomizer and pots were kept in moist chamber. Callose staining were performed after 5–7 DPI of incubation^67^. The infected leaves were incubated in 5 mL buffer [1 vol (water saturated phenol: glycerol: lactic acid: water in 1:1:1:1) +2 vol ethanol] at 65 °C temperature for 15–30 min. The leaves were transferred into fresh buffer for 25 h, rinsed 3 times each in ethanol and ddH_2_O. Stained in 150 mM K_2_HPO_4_ (pH 9.5) containing 0.01% aniline blue for 30 min, four washing was done in buffer (10 min each). The stained leaves were imaged under confocal microscope (Carl Zeiss’ LSM 510 META) with 20x Plan-apochromate lens. The excitation was done using diode laser 405 nm and emission was collected using band pass filter 470–500 nm.

### DAB staining

For *in situ* detection of H_2_O_2_ DAB staining was carried out using leaves of transgenic and control plants^68^. The sample were collected after 48 h of fungal spray and infiltrated under gentle vacuum with 1 mg mL^−1^ DAB containing 0.05% v/v Tween 20 and 10 mM sodium phosphate buffer pH 7.0. The reaction was completed within 4–5 h post inoculation after visualizing the brown precipitate at the surface of the leaves. Leaves were fixed and then boiled for 15 min in ethanol: acetic acid: glycerol (3:1:1). Bleaching solution was replaced and leaves were incubated until the chlorophyll was completely removed. Leaves were observed by light microscopy under bright field at 4X magnification.

### Measurement of H_2_O_2_

The H_2_O_2_ content of the leaves was measured spectrophotometrically after reaction with potassium iodide (KI)^69^ .The absorbance of the supernatant was measured at 390 nm from the spectrophotometer. The amount of hydrogen peroxide was calculated using a standard curve prepared with known concentrations of H_2_O_2_ (Supplementary Fig. 8a).

### SOD assay

Superoxide dismutase (SOD, EC 1.15.1.1) activity was measured by nitrotetrazolium (NBT) photochemical assay^70^. The total protein of plants were measured by Bradford^65^ method using bovine serum albumin (BSA, Sigma Aldrich, USA) as a standard (Supplementary Fig. 8b).

### Statistical analysis

Mean, standard error and number of replicates were used for statistical evaluation using GraphPad Prism 5. The statistical significance of differences between control and treated samples was tested by Dunnett’s test and unpaired Student’s t-test.

## Additional Information

**How to cite this article**: Singh, G. *et al*. Silencing of sterol glycosyltransferases modulates the withanolide biosynthesis and leads to compromised basal immunity of Withania somnifera. *Sci. Rep*. **6**, 25562; doi: 10.1038/srep25562 (2016).

## Supplementary Material

Supplementary Information
