# Supplementary material for: Silencing of sterol glycosyltransferases modulates the withanolide biosynthesis and leads to compromised basal immunity of Withania somnifera
Source: Sci Rep. 2016 May 5;6:25562. doi: 10.1038/srep25562 (PMC4857139; doi:10.1038/srep25562)
Supplement: Supplementary Information [file srep25562-s1.pdf]

Silencing of sterol glycosyltransferases modulates the withanolide biosynthesis and leads to compromised basal immunity of *Withania somnifera*

Gaurav Singh[1,2], Manish Tiwari[1,#], Surendra Pratap Singh[1], Surendra Singh[2], Prabodh Kumar Trivedi[1] and Pratibha Misra[1,*]

[1]Council of Scientific and Industrial Research - National Botanical Research Institute, Rana Pratap Marg, Lucknow-228001, Uttar Pradesh, INDIA

[#]Present address: Western Kentucky University, USA

[3]Banaras Hindu University, Varanasi, Uttar Pradesh, INDIA

***Corresponding author**

Pratibha Misra

Email: pratibhaflora@gmail.com; pratibhamisra@nbri.res.in

Tel: O, +91-522-2297919;

Fax: +91-522-2205836, +91-522-2205839

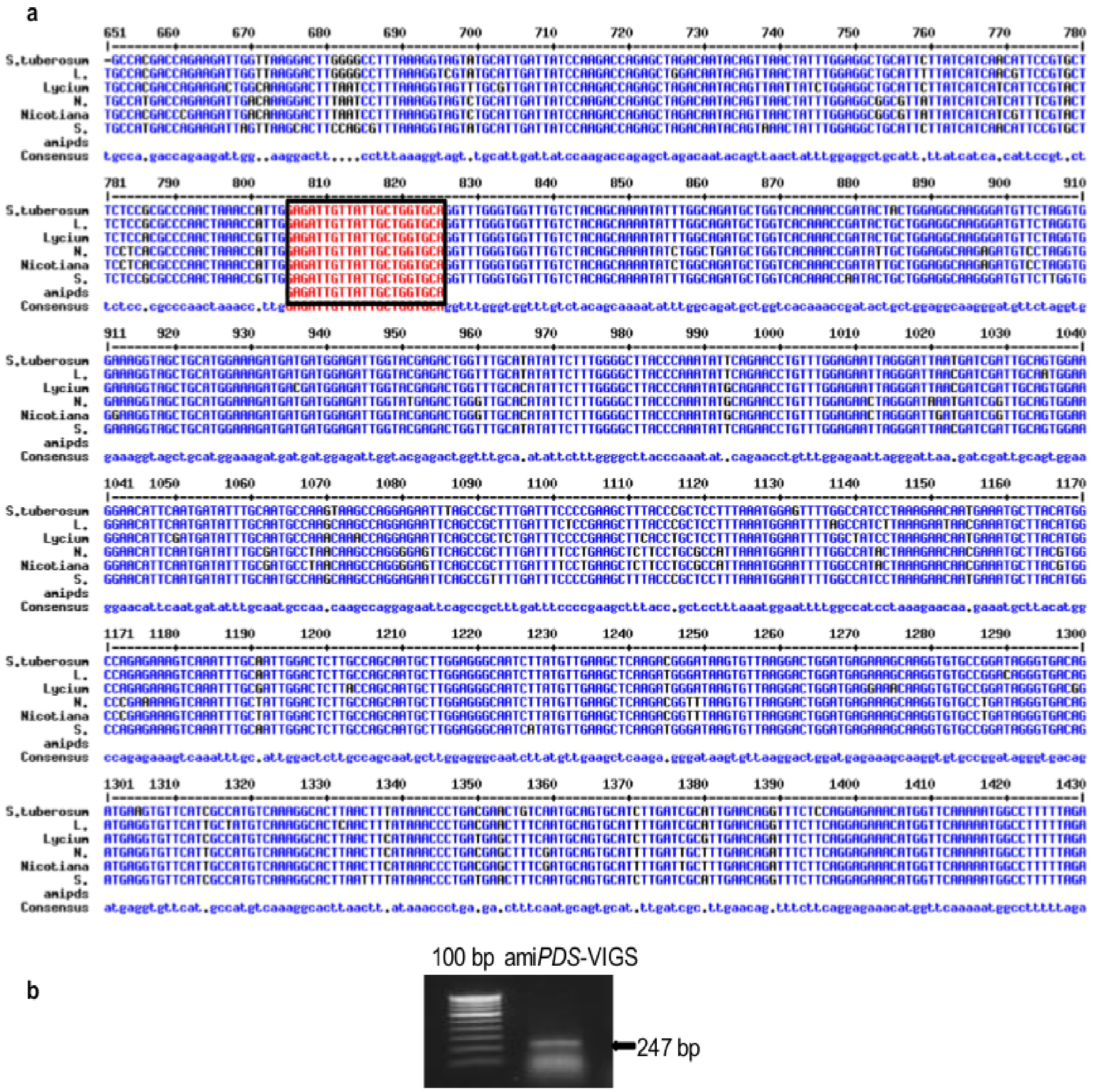

Supplementary Figure 1. Alignment of phytoene desaturase genes of Solanaceae and preparation of ami*pds*-VIGS construct.(a) Alignment of the phytoene desaturase gene of *N. benthamiana*, *S. nigrum*, *S. lycopersicum*, *L. esculentum*, *S. tuberosum*, through multialin online tool and 21 bp conserve region selected (red font black box) for preparation of primers. (b) Gel picture showed the cloned ami*pds* (247 bp) into VIGS vector.

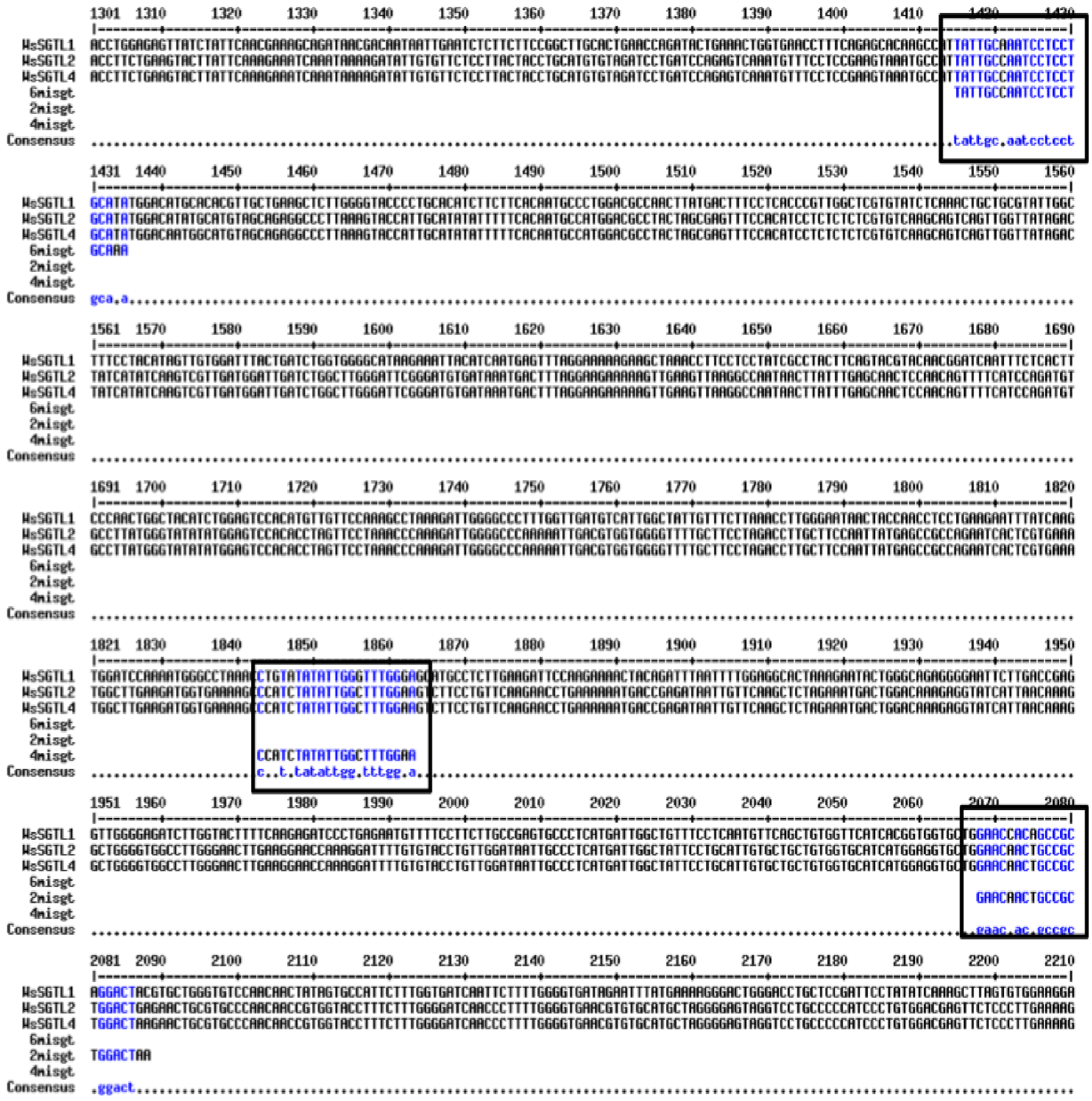

Supplementary Figure 2. amiRNA preparation for *WsSGTL* gene members of *W. somnifera*. Multiple alignments were performed by using of *SGTL1, SGTL2* and *SGTL4* of *W. somnifera* for the preparation of amiRNA through multalin tool. The highlighted regions showed the 21 bp sequence, selected for preparation of primers of amiRNA.

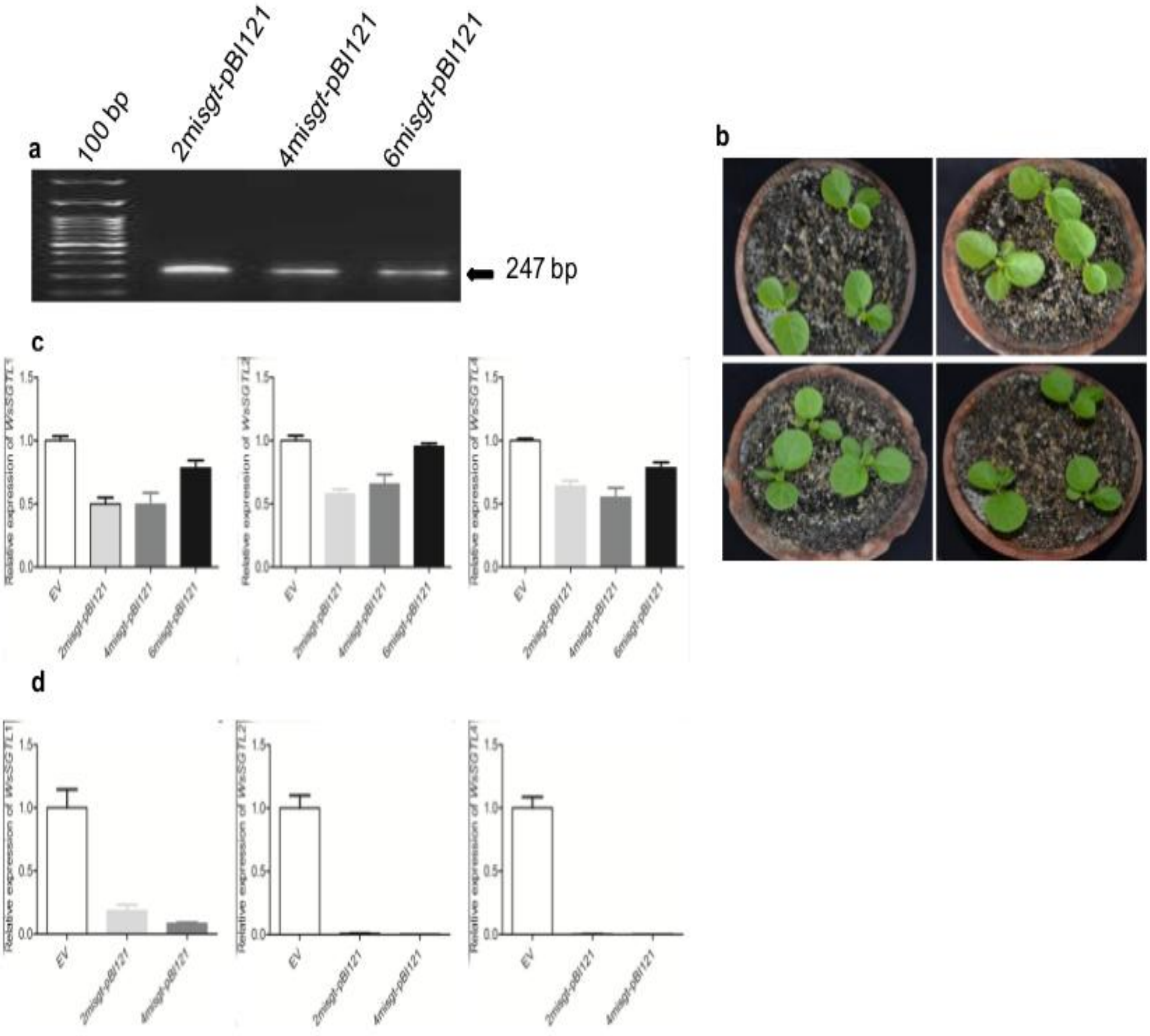

Supplementary Figure 3. Cloning and transformation of amiRNAs. (a) Gel picture of cloned 2mi*sgt* and 4mi*sgt* and 6mi*sgt* amiRNA into pBI121 vector (amplicon size 247 bp). (b) Stage for syringe infiltration in *Withania* i.e., 3 weeks old plants after seed germination. (c and d) qRT PCR of *WsSGTLs* members from the infiltered plant leaves by 2mi*sgt*-pBI121, 4mi*sgt*-pBI121 and 6mi*sgt*-pBI121 constructs after 48 h and 72 h.

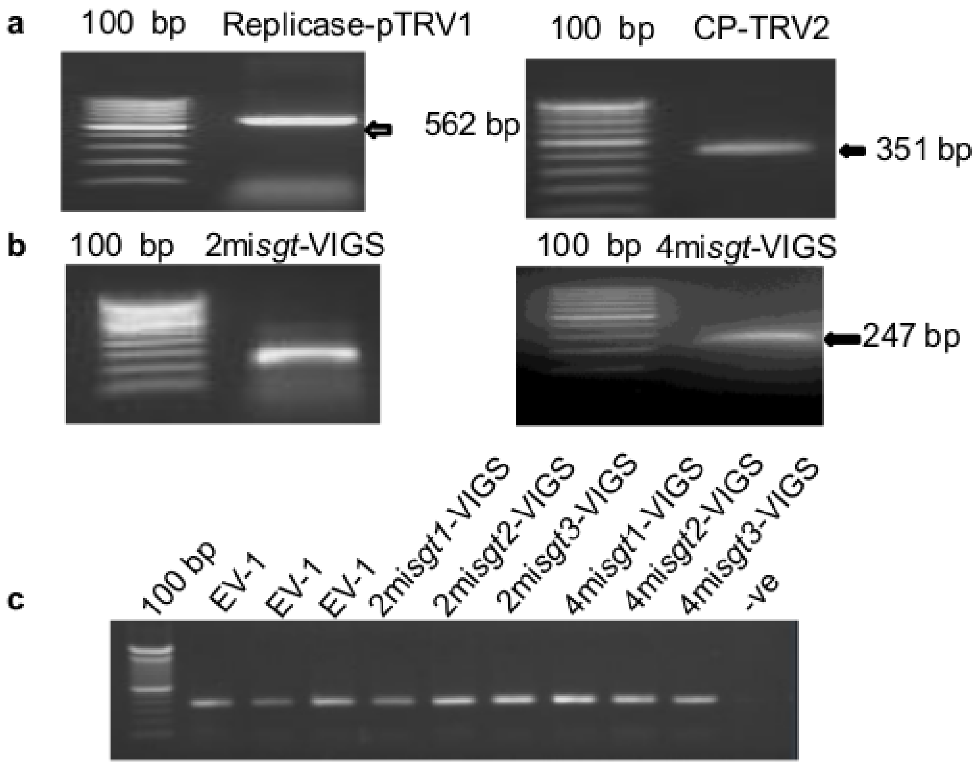

Supplementary Figure 4. aMIR-VIGS constructs of ami*sgt*s. (a and b) Colony PCR of positive VIGS constructs into GV 3101 strain of *Agrobacterium* by replicase, coat protein and by *WsSGTL* amiRNA specific primers. (c) Gel picture showed the confirmation of positive down-regulated lines of *W. somnifera* by coat protein specific primer after 3 weeks of the infiltration.

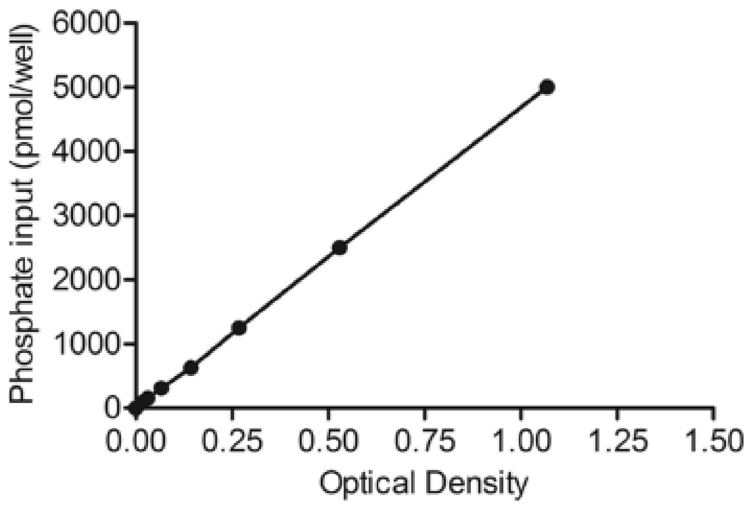

Supplementary Figure 5. **Standard curve preparation for SGT assay.** The slope of the linear regression line, 4683 pmol/OD, represents the amount of phosphate corresponding to a unit of absorbance at 620 nm.

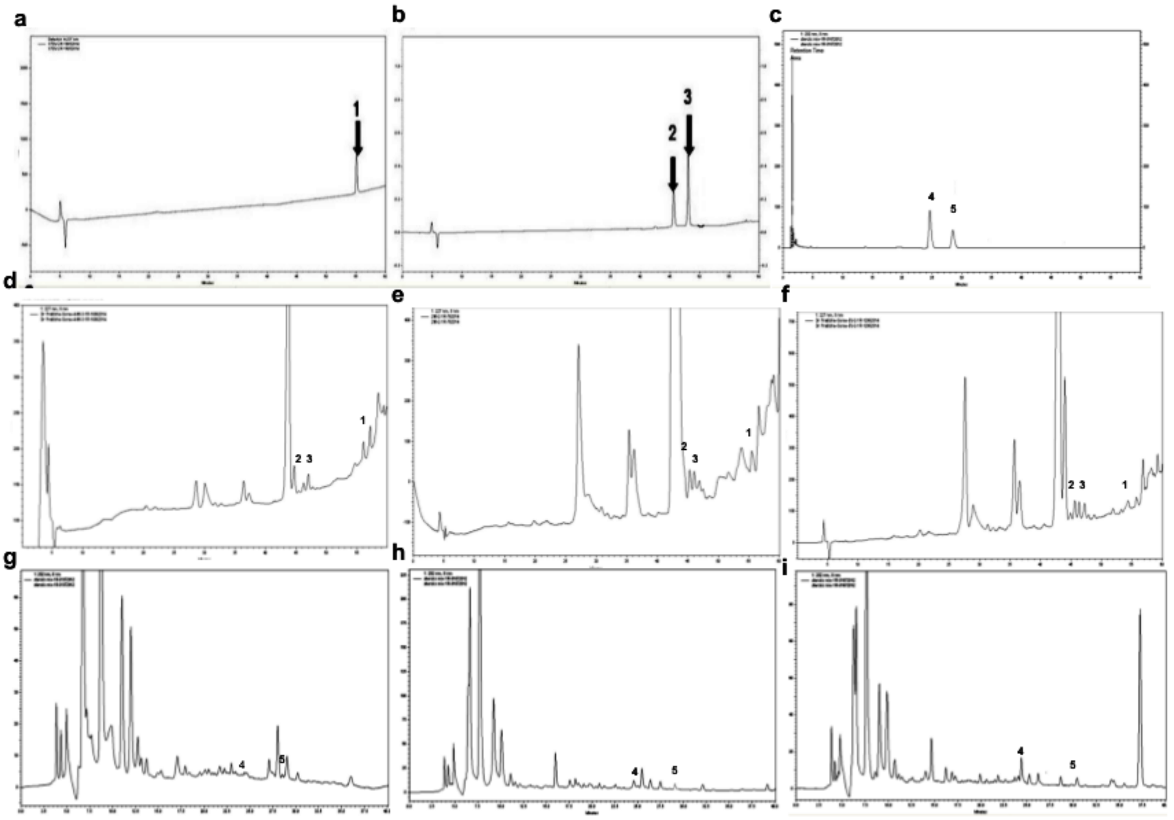

Supplementary Figure 6. HPLC chromatogram withanolides and sterols in EV and transgenic lines. (a, b and c) Standard HPLC chromatogram of 1) Withanoside V RT=55.12 (upper left), 2) withanolide A RT= 48.3 min, 3) withaferin A RT= 45.96 min (upper middle), 4) sitosterol RT= 28.48 and 5) stigmasterol RT= 24.6 (upper right). (d, e and f) HPLC Chromatogram of EV (central left), 2mi*sgt*-VIGS (central middle) and 4mi*sgt*-VIGS (central right) showing the relative abundance of different withanolides. (g, h and i) Chromatogram of the sitosterol and stigmasterol in the leaves of EV (lower left), 2mi*sgt*-VIGS (lower middle) and 4mi*sgt*-VIGS (lower right).

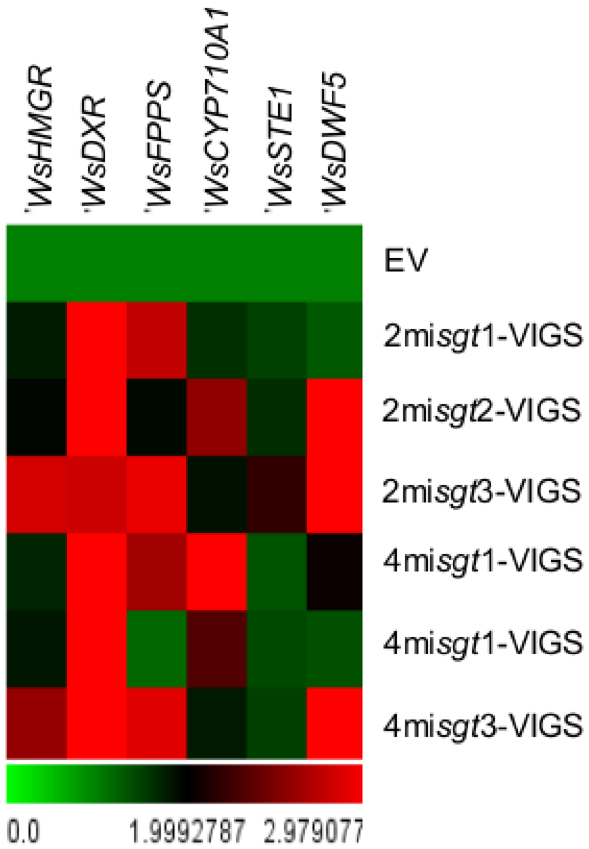

Supplementary Figure 7. Relative expression of important intermediate genes of withanolide biosynthesis pathway.Heat map showing the relative expression levels of MVA, MEP pathway genes in EV and leaves down-regulated lines. Data are means± SE of three biological replicate technical replicates using cDNA prepared from EV and transgenic plants. Expression levels of these genes were normalized to *Actin* and are represented in comparison with EV control

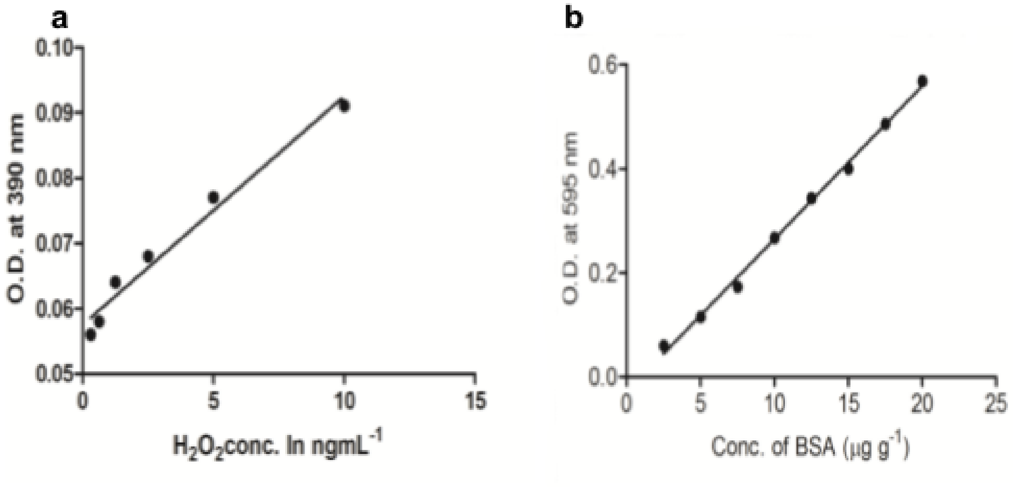

Supplementary Figure 8. Standard curve preparation for H_2O_2 and SOD. (a) Standard curve prepared by known concentration of hydrogen peroxide at 390 nm. (b) Standard curve preparation for Bradford assay by known concentration of bovine serum albumin (BSA) at 595 nm.

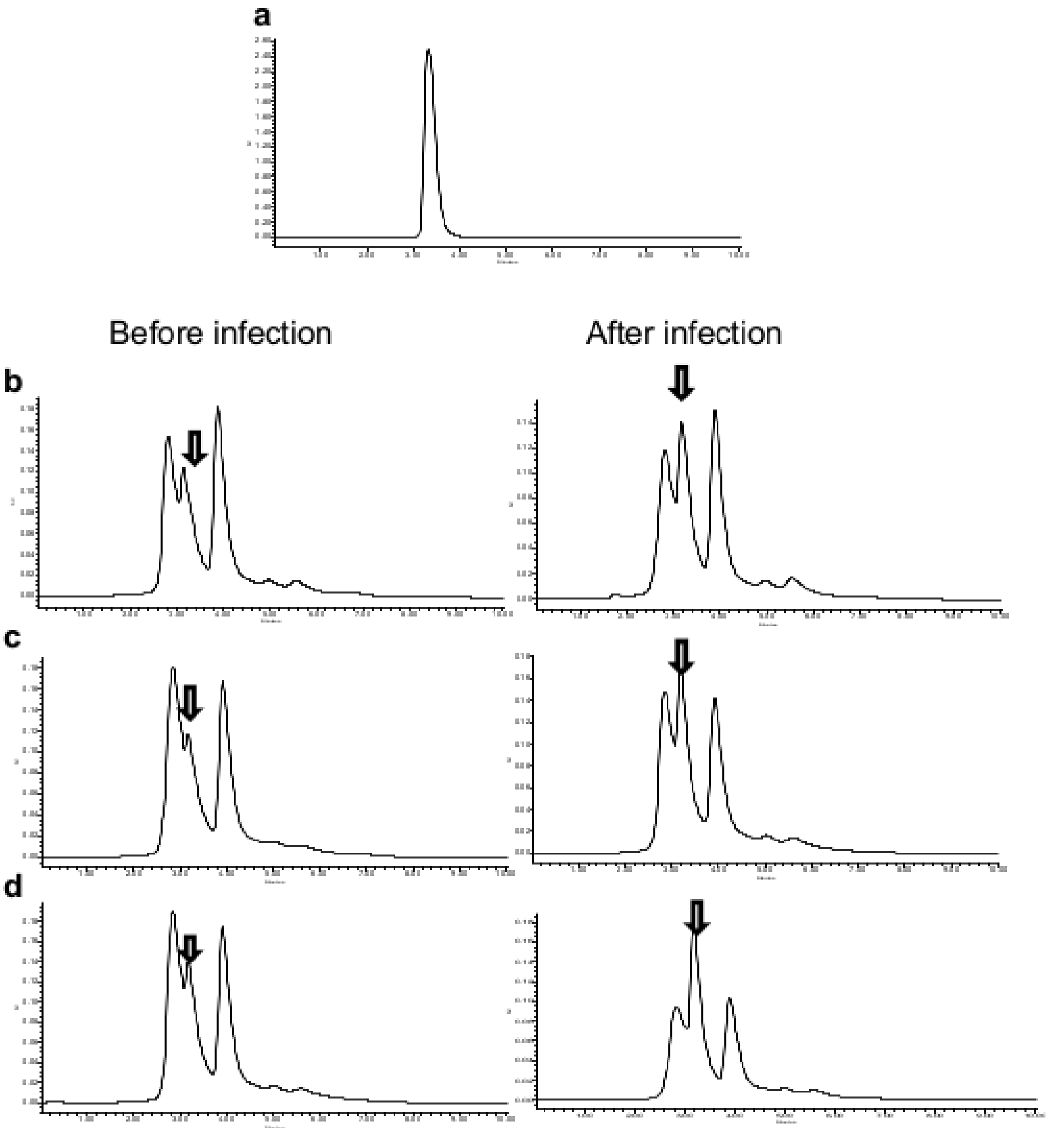

Supplementary Figure 9. Salicylic acid estimation in EV and transgenic lines before and after infection of *A. alternata.* (a) HPLC chromatogram of salicylic acid (SA) standards at RT= 3.3. (b, c and d) The SA estimation in the leaves of EV and transgenic lines before and after 7 days infection of *A. alternata* of *W. somnifera* with photodiode array detection (PDA).

Supplementary Table 1. Designed amiRNA primers for the *PDS* and *WsSGTL* members.

**Name**	**Sequences (5'-3')**
**ami*pds* F**	AAGATAGATCTAGATCTGACGATGGAAGGAGATTGTTATTGCTGGTGCA CATGAGTTGAGCAGGGTA
**ami*pds* R**	AAAATAGAGCTCGTGAAAGAAGGAGATTGTTATTGCTGGTGCA AAAGAAGAGTAAAAGCCATTA
**Ws*SGTL*2miF**	AAGATAGATCTAGATCTGACGATGGAAGGAACAACTGCCGCTGGACTAACA TGAGTTGAGCAGGGTA
**Ws*SGTL*2miR**	AAAATAGAGCTCGTGAAAGAAGGAACAACTGCCGCTGGACTAAAAAGAA GAGTAAAAGCCATTA
**Ws*SGTL*4miF**	AAGATAGATCTAGATCTGACGATGGAAGCCATCTATATTGGCTTTGGAACA TGAGTTGAGCAGGGTA
**Ws*SGTL*4miR**	AAAATAGAGCTCGTGAAAGAAGCCATCTATATTGGCTTTGGAAAAAGAA GAGTAAAAGCCATTA
**Ws*SGTL*6miF**	AAGATAGATCTAGATCTGACGATGGAAGTATTGCCAATCCTCCTGCAAACA TGAGTTGAGCAGGGTA
**Ws*SGTL*6miR**	AAAATAGAGCTCGTGAAAGAAGTATTGCCAATCCTCCTGCAAAAAAGAA GAGTAAAAGCCATTA

Supplementary Table 2. Primers used in the validation of this study.

**Primers**	**Sequences (5'-3')**
**CPP F**	CTGACTTGATGGACGATTCTT
**CPP R**	TGTTCGCCTTGGTAGTAGTA
**ReplicaseF**	CGGCACCCGATCTACAAGAA
**ReplicaseR**	GCCCACTCATCTAGCCCAAA
**RT- *PDS*F**	AGGTGGAAAGGTAGCTGCAT
**RT- *PDS*R**	GGCTGAATTCTCCTGGCTTG
**RT-*WsSGTL*F**	GCCGAGTGCCCTCATGATT
**RT-*WsSGTL1*R**	GTTGGACACCCAGCACGTAGTC
**RT-*WsSGTL2*F**	TGCAATTTGAAGTTCCGGGG
**RT-*WsSGTL2*R**	AGGGTTGCACATCTCCTCTC
**RT-*WsSGTL4*F**	GAACAACTGCCGCTGGACTA
**RT-*WsSGTL4*R**	CCTAGCATGCACACGTTCAC
**miR159-F1**	ATATCTCCTTCATAGCTCTAATG
**miR159-F1**	AAATAACACGCTAAACATTGCTTCG
**RT-*Actin (Act*)F**	AGATATTCAGCCTCTTGTCTGTG
**RT-*Actin (Act)*R**	ATTGAGCCTCATCACCAACATA
**RT-*WsPR1*F**	TGATGAGAAGCAATGGTATGACTAT
**RT-*WsPR1*R**	CGATCAGACATCAGTTGGAAGT
**RT-*WsDFSN*F**	TGCTGGTTTTTGCTACTGAGGCA
**RT-*WsDFSN*R**	CAGAAGCAACGGCGACGGAATC
**RT-*WsSPI*F**	ATGCCCGTCAAATTCATTAAGTTT
**RT-*WsSPI*F**	TCCTCCAGTCTCCAACAATCTA

**RT-*WsPR10*F**<br>**RT-*WsPR10*R**	AGTTGCTCATATAGAAGTCAAGTGT<br>TCCATCATAGTTCAATCTCCATTCA
**RT-*WsHMGR* F**<br>**RT-*WsHMGR* R**	GGAGTAGCAGGGCCGTTGTT<br>GCTTGTGCCGAACCTGACAC
**RT-*WsDXR* F**<br>**RT-*WsDXR* R**	CTCCGACCGCTTAGTAAATCCA<br>GCGAAGCATCGAGAGGAGTT
**RT-*WsFPPS* F**<br>**RT-*WsFPPS* R**	TCCAAACTGCCTCTGGACAA<br>GGCGATGGATAGGCAATGAG
**RT-*WsCYP710A1*F**<br>**RT-*WsCYP710A1*R**	CGGTCACCCTTTTGGGAAA<br>CGGCGGAGTTCCTTATGTTC
**RT-*WsSTE1*F**<br>**RT-*WsSTE1l*R**	ACAAGCCCTCCAGGAATATG<br>GGCAGTGCCACACGTTATAG
**RT-*WsDWF5*F**<br>**RT-*WsDWF5*R**	AGGACTGCCCAAGAAATCAT<br>TTCCACTGATCACGGTTCAT
